# Measurement of Tip Apex Distance and Migration of Lag Screws and Novel Blade Screw Used for the Fixation of Intertrochanteric Fractures

**DOI:** 10.1371/journal.pone.0170048

**Published:** 2017-01-13

**Authors:** Jesse Chieh-Szu Yang, Hsin-Chang Chen, Yu-Shu Lai, Cheng-Kung Cheng

**Affiliations:** 1 Division of Joint Reconstruction, Department of Orthopaedics, Taipei Veterans General Hospital, Taipei, Taiwan; 2 Department of Biomedical Engineering, National Yang-Ming University, Taipei, Taiwan; 3 Department of Orthopaedics, Taipei City Hospital, Taipei, Taiwan; 4 Orthopaedic Device Research Center, National Yang-Ming University, Taipei, Taiwan; University of Zaragoza, SPAIN

## Abstract

Fixation with a dynamic hip screw (DHS) is one of the most common methods for stabilizing intertrochanteric fractures, except for unstable and reverse oblique fracture types. However, failure is often observed in osteoporotic patients whereby the lag screw effectively ‘cuts out’ through the weak bone. Novel anti-migration blades have been developed to be used in combination with a lag screw (‘Blade Screw’) to improve the fixation strength in osteoporotic intertrochanteric fractures. An in-vitro biomechanical study and a retrospective clinical study were performed to evaluate lag screw migration when using the novel Blade Screw and a traditional threaded DHS. The biomechanical study showed both the Blade Screw and DHS displayed excessive migration (≥10 mm) before reaching 20,000 loading cycles in mild osteoporotic bone, but overall migration of the Blade Screw was significantly less (p ≤ 0.03). Among the patients implanted with a Blade Screw in the clinical study, there was no significant variation in screw migration at 3-months follow-up (P = 0.12). However, the patient’s implanted with a DHS did display significantly greater migration (P<0.001) than those implanted with the Blade Screw. In conclusion, the Blade Screw stabilizes the bone fragments during dynamic loading so as to provide significantly greater resistance to screw migration in patients with mild osteoporosis.

## Introduction

Intertrochanteric fractures are common injuries in the elderly and are often a consequence of sudden impact to an osteoporotic hip, such as that experienced during a fall [1]. Dynamic hip screw (DHS) is one of the primary choices for the fixation of intertrochanteric fractures (AO 31-A1). However, there is also an inherently failure risk with the use of a DHS, possibly down to the lag screw design. Madsen et al. [2] showed that 9% of patients receiving a DHS had a secondary fracture dislocation within a six month follow-up period, leading to complications such as varus malunion, lag screw cut-out or excessive lag screw sliding with medialization of the distal fracture fragment. In addition, postoperative fracture instability with secondary complications is frequently reported in severely osteoporotic patients [1–5]. In a sample of 178 intertrochanteric fractures treated with a DHS with a minimum of one year follow-up, Kim et al. [5] reported a complication rate in osteoporotic bone of greater than 50%. Poor bone quality is a major risk factor for intertrochanteric fractures, which could lead to an increased failure rate for implants or collapse and lag screw cut-out from the superior aspect in older patients [3,4].

Internal fixation of intertrochanteric fractures in osteoporotic bone is a challenge for orthopaedic and trauma surgeons. Osteoporosis reduces the thickness of cortical bone and increases the porosity of cortical and trabecular structures. This can subsequently lead to implant failure and joint collapse, as one the primary indicators of the mechanical stability of implants is bone quality. Various techniques and implants have been developed to enhance implant stability in osteoporotic bones and reduce lag screw cut-out. With the use of cement-augmented lag screws, Szpalski et al. [6] reported a higher success rate and lower incidence of cut-out complications in osteoporotic elderly patients with intertrochanteric fractures compared to those implanted with a conventional DHS. Lee et al. [7] investigated the use of polymethylmethacrylate (PMMA) cement augmentation in 108 elderly patients and indicated that the PMMA cemented DHS achieved superior fixation and patient outcome than a conventional DHS. However, PMMA cement augmentation has also been shown to induce additional complications, such as femoral head osteonecrosis, infection, subcapital femoral neck fracture, and partial destruction of the femoral head [8]. Wu et al. [9] also demonstrated that the failure modes of a cemented DHS were different from a conventional DHS, in that they were more likely to result in delayed union, nonunion and breakdown of the side plate. In order to avoid such complications with the use of a cemented DHS, several devices have been developed for stabilizing intertrochanteric fractures by adding an anchoring function to the lag screw [10,11].

**Fig 1:**
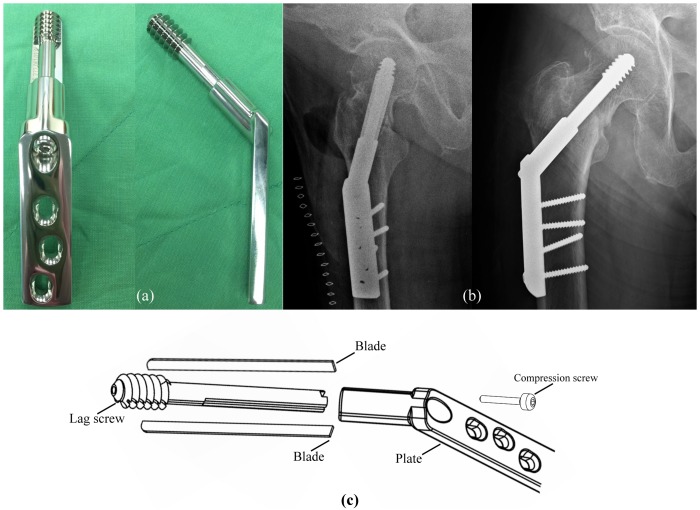
(a) Blade Screw DHS (ODRC Dynamic Hip Screw System, Chin Bone Corp., Taiwan; US FDA 510(k): K103015). (b) Radiographs of an 101 years-old woman showing fixation with a Blade Screw DHS post-operation. (c) The design and components of Blade Screw DHS.

To improve the fixation strength in intertrochanteric fractures, a bladed screw design was developed which combines a conventional lag screw shape with two anti-migration blades running along the length of the screw barrel (Fig 1) (Blade Screw; ODRC Dynamic Hip Screw System, Chin Bone Corp., Taiwan; US FDA 510(k): K103015). The addition of these longitudinal blades provides a number of advantages in terms of potentially reducing the risk of cut-out. Inserting the two blades into femoral head through the lateral cortical compacts the surrounding cancellous bone structure. After insertion, any movement of the femoral head would act to further compact the cancellous bone in the region of the blade surface. Also, the increased lateral surface area offered by the blades achieves the previously mentioned anchoring function. The increased support surface could theoretically decrease the rate of lag screw migration, which is important in improving fragment fixation and postoperative results. Therefore, the purposes for this study was 1) to compare lag screw migration and cut-out between the Blade Screw and a conventional DHS through *in vitro* gait simulation, and 2) to identify signs of early lag screw migration on two-dimensional radiographs of intertrochanteric fractures treated using the Blade Screw and a DHS.

## Materials and Methods

### In vitro biomechanical tests

**Fig 2:**
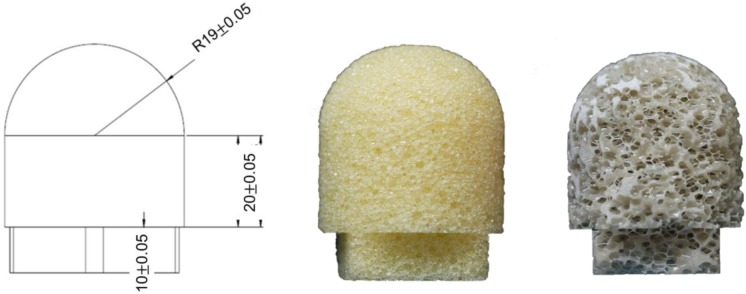
(a) Size of a test block; (b) Cellular rigid polyurethane foam (Type 1522–11) used to simulate a mild osteoporotic bone; (c) Open-cell rigid polyurethane foam (Type 1522–524) used to simulate a severely osteoporotic bone.

Two grades of commercially available synthetic bones were tested in this study. An open-cell rigid polyurethane foam with a density of 0.09 g/cm^3^ (Type 1522–524, Pacific Research Inc., Vashon, Washington, USA) represented the homogeneous and uniform material properties of severely osteoporotic cancellous bone. A cellular rigid polyurethane foam (Type 1522–11, Pacific Research Inc., Vashon, Washington, USA) was used to simulate mild osteoporotic bone. The polyurethane foams were purchased in blocks and molded to the shape of a femoral head with a radius of 19 mm, height of 20 mm and a base thickness of 10 mm (Fig 2). In total 36 femoral head models were created, 18 from each grade of sawbone. These were subsequently tested grouped into two categories for testing on the two lag screws: the Blade Screw and conventional DHS (DHS Screw, Synthes, USA). 9 sawbones from each grade were tested on each screw. The barrel of the Blade Screw has a length of 120 mm and an outer thread diameter of 12.7 mm. The blades themselves run along the barrel with a length of 110 mm and a width of 4.4 mm. The DHS has a length of 120 mm and an outer thread diameter of 12.7 mm.

The lag screws were implanted into a central position in the femoral head test models. Before inserting the screw, a pilot hole was gimleted into the femoral head using a 10.5 mm drill bit. The cannulated lag screw was then inserted through the pilot hole, and the tip of screw was kept at distance of 10 mm from the apex of the femoral head. Accurate screw insertion was confirmed using a custom-made guide to ensure the proper distance and location of the lag screw within the femoral head. The femoral head test models were placed in a polished steel shell with a thickness of 6 mm which provides a rigid and spherical interface for the dynamic gait simulations.

**Fig 3:**
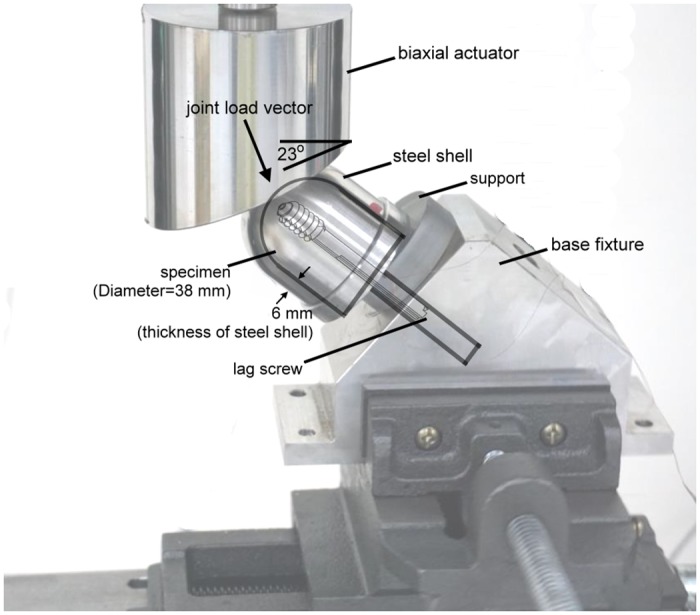
A femoral head test model was fixed in the HIPS system and subjected to multi-axial loading.

A hip implant performance simulator (HIPS) system was created to evaluate lag screw migration in each femoral head test model after multi-axial loading that simulates walking conditions (Fig 3) [12]. The upper fixture of the HIPS system was fitted with a load cell for recording forces on the fixture. A 23° inclined block was also attached to the upper fixture. The lower fixture of the HIPS system simulated a femoral shaft with its anatomic axis aligned perpendicular to the horizontal plane of the material testing machine (MTS, BOSE 3510-AT). The superior surface of the lower fixture was oriented at 40° to the anatomic axis of the simulated femoral shaft to resemble an intertrochanteric fracture line. A polyethylene ‘support’ plate was also attached to the lower fixture to reproduce the constraint characteristics of a reduction in an intertrochanteric fracture [13]. The femoral head test model implanted with a lag screw was fixed to the superior surface of the lower fixture. In addition, the bottom of the femoral head test model is a cuboid shape, which sits into a corresponding cuboid attachment at the base of the steel shell.

The test structure was subjected to sinusoidal loading ranging from 5 N to 1,450 N at a frequency of 1 Hz, simulated walking conditions [12]. To simulate the hip joint motions during gait, concurrent flexion-extension and abduction-adduction motions were superimposed by sinusoidal rotation of the 23° inclined loading block. According to Ehmke et al. [12], the 23° inclination could account for an 18° resultant joint load vector and a 5° valgus angle of the femoral shaft axis. Excessive gait kinematics of a limb was simulated by ± 75° rotation of the actuator, resulting in a 45° arc of flexion-extension and a 17° arc of abduction-adduction. The test was stopped when axial displacement of the actuator reached 10 mm, defined as excessive migration, or loading reached 20,000 cycles [12].

Four marks were placed 90° apart on the surface rim of the steel shell. Spatial migration of the femoral head test model in relation to the lag screw was continuously recorded using a pure video-based tracking and analysis software (Tracker Video Analysis and Modeling Tool) [14]. An independent t-test was used to compare differences in displacement of the femoral head (DFH), displacement of the distal lag screw tip (DDLS), varus collapse (VC), and rotation around the neck axis (RN).

### Clinical follow-up and the measurement of tip apex distance

From May 2013 to July 2014, intertrochanteric fracture fixation was performed by the same surgeon on twenty female patients: 11 fixed with a Blade Screw and 9 fixed with a conventional DHS. Inclusion criteria were low bone mineral density (T-score < = -2.0), stable intertrochanteric fractures (AO/OTA 31-A1/2) and a minimum of 3 months follow-up. Additional exclusion criteria were incomplete chart records, radiographs not meeting diagnostic standards and loss to follow-up. Both patient groups presented classifiable intertrochanteric fractures (AO/OTA 31-A1) with a mean patient age of 76 years (range, 53–101) for the Blade Screw group and a mean age of 80 years (range, 59–93) for the DHS group. No patients presented intraoperative complications, infections or intraoperative or postoperative femoral fracture around the screw tip during a 3-month follow-up period. The bone mineral density (BMD) of all patients was measured and evaluated using a T-score. As lag screw cut-out through the femoral head generally occurs after weight bearing, the anteroposterior (AP) and lateral radiographs of each patient were collected immediately after surgery and at 3 months follow-up. The distance from the tip of the screw to the apex of the subchondral bone (tip-apex-distance, TAD) was measured with AutoCAD software to determine the position of the lag screw within the femoral head on the AP and lateral radiographs [15, 16]. The TAD value was considered as a predictor of lag screw cut-out and has been suggested to be kept less than 25 mm to prevent DHS cut-out failure [15,16]. An independent t-test analysis was used to compare the inter-group differences in age and BMD. A two-factor mixed design ANOVA was used to assess group differences in TAD values immediately after surgery and at 3 months follow-up.

The study protocol was approved by the Institutional Review Board (IRB) of National Yang-Ming University, Taiwan on 20 February 2015 (IRB No.: YM103107WE). All patients provided informed written consent prior to their clinical records being accessed and published and prior to being included in this study.

## Results

### In vitro biomechanical testing in mild osteoporotic bone model

**Fig 4:**
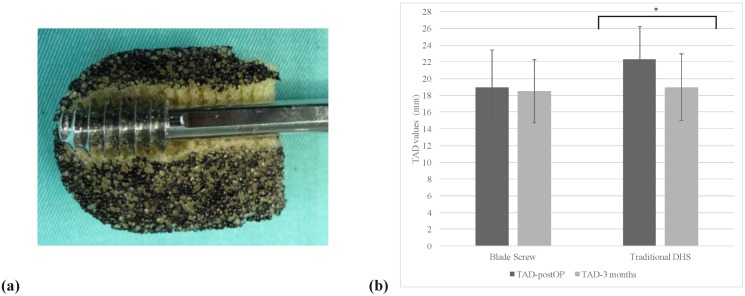
(a) Specimen after lag screw excessive migration (≥10 mm); (b) TAD values of the Blade Screw and traditional DHS groups immediately after surgery and at 3-months follow-up.

In the mild osteoporotic femoral models, both screw types demonstrated excessive migration (≥10 mm displacement) before reaching 20,000 loading cycles (Fig 4a). The mean number of cycles achieved by the Blade Screw and traditional DHS were 11,000±409 and 6,200±250 cycles respectively. The DFH, DDLS, VC, and RN of the Blade Screw group were all significantly smaller than those recorded from the traditional DHS group at the point of excessive migration (p ≤ 0.03; Table 1).

**Table 1:** Displacement of the femoral head (DFH), displacement of the distal lag screw (DDLS), varus collapse (VC), and femoral head rotation around the neck axis (RN) of the Blade Screw and traditional DHS.

	DFH (mm)	DDLS (mm)	VC (°)	RN (°)
**Blade Screw**	9.13±1.39	0.31±0.16	0.50±0.61	1.61±1.96
**Traditional DHS**	11.68 ± 0.41	1.96 ± 0.95	10.61 ± 2.19	16.93 ± 2.52
**p-value**	0.03	0.003	<0.001	<0.001

p<0.05 indicates a significant different between the two lag screws.

### In vitro biomechanical testing in severe osteoporotic bone model

In the severe osteoporotic bone models, both screw types demonstrated excessive migration before reaching 1,000 loading cycles. The mean number of loading cycles achieved by the Blade Screw and traditional DHS were 642±26 and 125±11 respectively. At the point of excessive migration, the mean DFH of the Blade Screw was 9.44±0.99 mm, which was significantly less than the traditional DHS (11.26±0.77 mm). Because the bone models were destroyed after relatively few loading cycles, the mean DDLS, VC, and RN could not be accurately recorded or analyzed.

### Clinical follow-up and the measurement of tip apex distance

The mean BMD of the Blade Screw group was 0.732±0.1224 g/cm^2^ (T score = -2.8) and the mean of traditional DHS group was 0.871±0.2572 g/cm^2^ (T score = -2.7). The difference in BMD between the two groups was not significant. Also, no significant difference was detected in mean TAD between the two groups immediately after surgery and at 3-months follow-up. In addition, there was no significant intra-group variation in TAD seen in the Blade Screw group either post-surgery or at follow-up (P = 0.12). However, the mean TAD in the traditional DHS group immediately after surgery was significantly larger than at 3-months follow-up (P<0.001). By using a post hoc power analyses based on the repeated-measures differences in TAD values, the results demonstrated 84% power in detecting time differences at a significance level of 0.05 (Fig 4b).

## Discussion

This study compared lag screw migration between two lag screw designs, the Blade Screw and a traditional DHS, implanted in osteoporotic bone models. In the mild osteoporotic sawbone models, the Blade Screw group withstood a greater number of loading cycles before reaching the point of excessive migration of the implant (≥10 mm). This indicates a greater resistance to implant migration through the bone. Kouvidis et al. used similar testing protocols to investigate the number of loading cycles to implant failure in single and dual lag screw systems and showed that the average number of cycles leading to cut-out was 6,638 and 10,054, respectively [17]. In the current study, the amount of loading cycles withstood by the traditional DHS and Blade Screw approximate those of single and dual lag screw systems, signifying the superior cut-out resistance of the Blade Screw. In addition, indicators of lag screw migration (DFH, DDLS, VC, RN) with the Blade Screw were all significantly less than with the traditional DHS (Table 1). This indicates the greater fixation strength of the Blade Screw construct under multidirectional dynamic loading. In addition, the low VC and RN of the Blade Screw also demonstrate its superior resistance to rotation, which is particularly important when the fracture line is perpendicular to the femoral neck. Rotation of a lag screw in this type of fracture would destabilize the femoral head fragments and result in varus collapse under multi-directional loading [12].

In the severely osteoporotic bone model, both lag screws failed to achieve good bone purchase. Since engagement of the cortex was neglected, the structure of the open-cell rigid polyurethane foam was destroyed before the 1,000^th^ loading cycle. Therefore, it was not possible to adequately evaluate lag screw migration using the current method. However, the Blade Screw did sustain a greater number of loading cycles before the point of excessive implant migration. Displacement of the femoral head was also less than in the traditional DHS model. The two longitudinal blades on the Blade Screw enlarge the contact surface between the lag screw and bone in the superior-inferior direction, resulting in additional resistance to rotation of the femoral head around the screw.

In addition to the sawbone implantation models, 20 patients exhibiting intertrochanteric fractures were also treated with lag screws; 11 with a Blade Screw and 9 with a DHS. Immediately after surgery and at 3-months follow-up, the mean TAD of both groups was less than 25 mm, indicating a low risk of lag screw cut-out in all patients [15–20]. Although there was no significant inter-group difference in mean TAD immediately after surgery, a significant reduction in mean TAD was observed in the DHS group at 3-months follow-up. This may suggest that the fixation of the lag screw in the femoral head was sufficient enough to limit the relative motion. Furthermore, the postoperative initial stability of intertrochanteric fractures is important for reducing complications associated with long term immobilization and also allows for early rehabilitation [21]. The steady position of the lag screw within the femoral head observed in the Blade Screw group corresponds with the biomechanical test results. However, further investigation with a long-term follow-up period would be necessary.

There are some limitations to the methods used in this study that should be noted. First, the femoral head bone samples were synthetic bones made of rigid polyurethane foam with isotropic mechanical properties. Although these models might not fully reproduce the biomechanical properties of human bone, these are standardized sawbones that have been shown to provide consistent material properties similar to a human cancellous bone [10, 22]. Synthetic bones are the most reliable and reproducible choice for biomechanical testing in the absence of cadaveric bone. They also offer consistency across test samples. Secondly, the in-vitro biomechanical test might not represent the true fracture properties and physical loading of an individual. In addition, micromotion between the polyurethane femoral head and steel shell may affect the accuracy of recording true screw migration. However, a retrospective clinical evaluation of the patients in this study could help determine if the favorable biomechanical test results of the Blade Screw are dependent on clinical practice. Third, the post-surgical follow-up period was short, and possibly not long enough to truly determine the incidence of implant failure. Studies have shown screw cut-out to occur between 1 to 6 months after implantation [23,24]. A longer term study may offer more accurate results on the risk of screw cut-out and other post-surgical complications.

## Conclusion

Compared to the traditional DHS, the Blade Screw provides significantly greater resistance to lag screw migration and cut-out in low density bones. The findings of this study show that the Blade Screw design has the advantage of improving the postoperative fixation in osteoporotic bones by securing the fracture fragments during dynamic loading.
